# A spatiotemporal meta-analysis of HIV/syphilis epidemic among men who have sex with men living in mainland China

**DOI:** 10.1186/s12879-018-3532-8

**Published:** 2018-12-12

**Authors:** Rong Fu, Jinkou Zhao, Dan Wu, Xiayan Zhang, Joseph D. Tucker, Meiwen Zhang, Weiming Tang

**Affiliations:** 1 Dermatology Hospital, Southern Medical University, Guangzhou, 510095 Guangdong China; 2 University of North Carolina Project-China, Guangzhou, China; 3 SESH (Social Entrepreneurship to Spur Health) study group, Guangzhou, China; 4 Technical Advice and Partnership Department, The Global Fund to Fight AIDS, Tuberculosis and Malaria, Geneva, Switzerland; 5 Shijingshan District Center for Disease Control and Prevention, Beijing, China; 6 Claremont Graduate University, Claremont, CA USA

**Keywords:** Men who have sex with men (MSM), HIV, Syphilis, Infection, Spatiotemporal meta-analysis

## Abstract

**Background:**

Geographic differences in HIV, syphilis and condomless sex among men who have sex with men in China remained unknown. We aimed to elucidate these spatiotemporal changing patterns in China.

**Methods:**

We conducted a spatiotemporal meta-analysis. We searched four databases for studies conducted between 2001 and 2015. We included studies that reported original data of HIV/syphilis prevalence in China, the study’s area/province, and period of data collection. We grouped studies into six regions and four time periods. We examined the changing patterns of national and regional prevalence of HIV, syphilis and condomless sex.

**Results:**

Search results yielded 2119 papers, and 272 were included in the meta-analysis. The sample sizes of the studies ranged from 19 to 47,231. National HIV prevalence increased from 3.8% (95% CI 3.0–4.8) in 2001–07 to 6.6% (5.6–7.7) in 2013–15. In most regions, the rise occurred before 2010 and the HIV prevalence remained relatively stable afterwards, except for the Northwest which showed a considerable increase since 2008. National syphilis prevalence decreased from 12.3% (10.2–14.9) in 2001–07 to 7.1% (5.6–8.9) in 2013–15.

**Conclusions:**

The trends of HIV and syphilis infections have been effectively curbed in MSM in most regions of China. Continuous efforts, particularly promotion of condom use, are needed to further reduce these infections.

**Electronic supplementary material:**

The online version of this article (10.1186/s12879-018-3532-8) contains supplementary material, which is available to authorized users.

## Background

Men who have sex with men (MSM) are a predominant group of HIV vulnerable populations and have been at substantial risk of HIV infection since the 1980s [1, 2]. A global epidemiologic study found disproportionately high HIV prevalence among MSM, with prevalence estimates from 3.0% in Middle East and North Africa to 25.4% in the Caribbean [2]. Many meta-analyses have been done to examine HIV epidemic trends and high-risk behaviors in Chinese, the results were similar to other low- and middle-income countries (LMIC),, reporting a sharp increase in HIV prevalence among Chinese MSM across the country since the early 2000s, [3–6] and national HIV prevalence in MSM increased from 1.8% in 2000 to 8.0% in 2015 [4, 7]. However, these studies have not linked the temporal trends of HIV epidemic with spatial patterns at regional level, which could not prove if there were disparity and changing trends of HIV prevalence among these regions.

Compared with other sexually transmitted infections (STIs), syphilis proved to be most frequent co-infection in HIV-positive MSM [8]. Though STIs had been virtually eliminated in 1964 among the whole population, national surveillance data from sentinel site network witnessed a 110-fold increase in syphilis diagnosis from 0.2 cases per 100,000 individuals in 1989 to 22 cases per 100,000 individuals in 2008 in China [9–11]. MSM, in particular, were one of the most vulnerable groups to such a burden, with syphilis prevalence increased from 9.1% in 2001–2008 to 14.3% in 2015 [12, 13]. A clear parallel to increasing HIV-syphilis co-infection explained that syphilis boosts the higher risk of HIV transmission, which may imply greater odds of HIV infection among MSM in China [5, 13, 14].

Apart from syphilis infections, Chinese MSM who were having condomless anal sex also were at higher risk for HIV infection [15, 16]. Condomless sex prevailed among high school and college MSM students [17]. In the worst-hit area like Yunnan province, condomless sex in MSM, which was greatly affected by sociodemographic and other sexual behavior factors, remained at a high level through some effects of HIV/AIDS intervention had been observed [18].

Knowing the spatiotemporal patterns of diseases is essential for effective resource allocation in any healthcare system. A 61-cities study reported the nationwide disproportionate geographical distribution of HIV prevalence among MSM [14]. These studies indicated that Southwestern China has been the most affected region, where HIV prevalence reached around 11.0%, compared with other regions with prevalence ranging from 3.5 to 4.8% in 2010 [4, 5]. Data from surveillance sites also found that HIV epidemic in MSM was spatially clustered in metropolis such as Beijing, Shanghai, Guangzhou, and municipalities and provincial capitals such as Chongqing and Chengdu [19]. However, all of these data were based on sentinel surveillance sites in 107 big cities of China, [19] which have better healthcare service and capacities. It lacks evidence about nationwide spatiotemporal patterns of HIV and syphilis among MSM and limited our ability to provide tailored services to MSM. Since HIV infection has become a chronic disease for the success of antiretroviral therapy [20], we therefore used prevalence rate to assess HIV infection and syphilis infection. We selected the time period between 2001 and 2015 as our study period based on the policy and guideline changes related to MSM and HIV/AIDS care and treatment: In 2008, the China CDC conducted the first national level survey among MSM in 61 cities, and China start to pay attention to the HIV/AIDS problem since then, and before this time period, very limited data are available for Chinese MSM. *AIDS Diagnosis and Treatment Guidelines* established by Chinese Medical Association Infectious Diseases Society AIDS Group in 2011, *12th Five-Year Plan of Action for China’s Containment and Prevention of AIDS* announced by Office of the State Council in 2012 and start to implement since 2013 [21–24] In this study, we defined condomless sex as sex without condoms and unprotected sex as having sex without a condom in an environment of heightened HIV and STI risks, which is in light of the language shifting of US CDC in 2014 [25]. We referred any male partners to casual male partner and regular male partner, and a casual male partner means he may have casual sex for a limited time or part-time, without necessarily demanding or expecting the extra commitments.

In this study, we conducted a meta-analysis and spatiotemporal analysis of the existing literature, and evaluated the trends of HIV infection, syphilis infection, and condomless sex among Chinese MSM between 2001 and 2015, with an aim of informing policymakers about channeling healthcare resources to provide improved care for the regions with the highest HIV and/or syphilis disease burdens.

## Methods

### Search strategy and selection criteria

We used meta-analysis and spatial analysis to assess trends of HIV, syphilis, and condomless sex, so this manuscript is not a typical systematic review and meta-analysis and we did not follow the formatting guidelines. Three investigators (RF, XZ and MZ) systematically reviewed published peer-reviewed research articles by searching PubMed, EMBASE, Cochrane, China National Knowledge Infrastructure (CNKI) and Wanfang from January 1, 2001, to June 8, 2016. After defined the population and disease, we consulted experts in this field, reviewed articles and journals in the same theme using meta-analysis and systematic review, and searched the subject terms in the above databases. The keywords (“Human immunodeficiency virus”, “HIV” OR “Acquired Immune Deficiency Syndrome”, “AIDS” OR “syphilis”) AND (“homosexual” OR “gay” OR “men who have sex with men” OR “MSM”) AND (“China” OR “Chinese”) were used in the database search. Additional studies were also identified by a manual search on the reference list of the published studies. The review was conducted and reported in accordance with PRISMA guidelines issued for Systematic Reviews and Meta-analysis in 2009 (Additional file 1).

We included studies which were: (1) conducted in mainland China (Hong Kong Special Administrative Region, Macao Special Administrative Region, and Chinese Taipei were not included); (2) conducted between 2001 and 2015; (3) published in Chinese or English language; (4) cohort or cross-sectional studies with original data of HIV/syphilis prevalence with information on the study’s area/province, period of data collection, and sample size. We excluded studies which: (1) lacked key information (as listed in inclusion criteria); or (2) were duplicated studies.

### Data analysis

We extracted the following data from all included studies: study location, year of publication and study period, sample size, prevalence of HIV and syphilis, and rates of high-risk behaviors (condomless sex with male partner in past one month, condomless sex with female partner in past 3 months, and condomless sex with casual male partner in past 3 months) and weighted each study equally. All studies were grouped according to study region and study period. We categorized study locations into six regions: North (Beijing, Tianjin, Hebei, Shanxi), Northeast (Liaoning, Jilin, Heilongjiang, Inner Mongolia), Northwest (Shaanxi, Gansu, Qinghai, Ningxia, Xinjiang), East (Shanghai, Jiangsu, Zhejiang, Anhui, Fujian, Jiangxi, Shandong), Southwest (Sichuan, Guizhou, Yunnan, Chongqing, Tibet) and South-central (Henan, Hubei, Hunan, Guangdong, Guangxi, Hainan). We categorized study periods into four periods of time: 2001–2007, 2008–2010, 2011–2012 and 2013–2015.

We conducted a meta-analysis to summarize HIV prevalence, syphilis infection prevalence, and condomless and unprotected sex rate by region and period using the ‘meta’ package in R version 3.3.1. Heterogeneity was evaluated using the I^2^ statistic, which ranged from 0 to 100%. If I^2^ > 50% then random effect model was used, otherwise fixed effect model. We then used ArcGIS 10.2 software (ESRI Inc., Redlands, CA, USA) to visualize HIV and syphilis prevalence on one map of China.

## Results

We identified 2119 articles in our search. Of these, 1476 were excluded after screening their titles and 230 were excluded after screening their abstracts (137 were a Ph.D./Master thesis, 46 were conference presentations, and 47 were reviews). Thus, 413 articles remained for full-text review. Of the 413 articles, 141 were excluded because the HIV and syphilis prevalence (*n* = 90), study period (*n* = 25), or study site (*n* = 12), were not included. We also excluded 14 duplicate studies. 272 papers were included in the final data analysis (Additional file 1 and Additional file 2).

Characteristics of the 272 studies are presented in Table 1 and meta-analyses of HIV and syphilis prevalence stratified by 6 geographical regions are shown in Additional file 3. Overall, 83 out of 272 (25.2%) studies reported data from the East region. Nearly half of the surveys were conducted between 2008 and 2010 (42.8%, *n* = 146) and more than half were published in 2013–2016 (59.6%, *n* = 162). In survey years 2008–2010, the number of studies in the Southcentral region (47) almost four timed that in Northwest region [12]. The sample sizes of the included studies ranged from 19 to 47,231, and 33 of them had 101–250 participants and 93 had 251–500 participants. Of the 272 studies, over 80% (*n* = 228) reported HIV prevalence, and 176 (64.7%) reported syphilis prevalence. There were 82 (30.1%), 48 (17.6%), and 64 (23.5%) studies that reported condomless sex with any male partners in the past one month (P1M), condomless sex with female partners in the past three months (P3M), and unprotected sex with casual male partners P3M, respectively. We reported condomless sex with female partners P3M on the premise that many Chinese gay men marry unknowing heterosexual partners due to family and societal pressure [26].

**Table 1:** Results and summary of study characteristics (*N* = 272)

Study Characteristics	*n*	%
Language		
Chinese	172	63.2
English	100	36.8
Region		
East	83	25.2
Southcentral	74	22.4
North	58	17.6
Southwest	49	14.8
Northeast	43	13.0
Northwest	23	7.0
Published year		
2001–2007	16	5.9
2008–2010	39	14.3
2011–2012	55	20.2
2013–2016	162	59.6
Study period		
2001–2007	42	15.4
2008–2010	146	53.7
2011–2012	108	39.7
2013–2015	45	16.5
Sample size		
1−100	9	3.3
101–500	126	46.3
501–1000	71	26.1
1001-	66	24.3
HIV infection description	228	83.8
Syphilis infection description	176	64.7
Condomless sex with male partner in past 1 month	82	30.1
Condomless sex with female in past 3 months	48	17.6
Unprotected sex with casual male partners in past 3 months	64	23.5

**Fig. 1:**
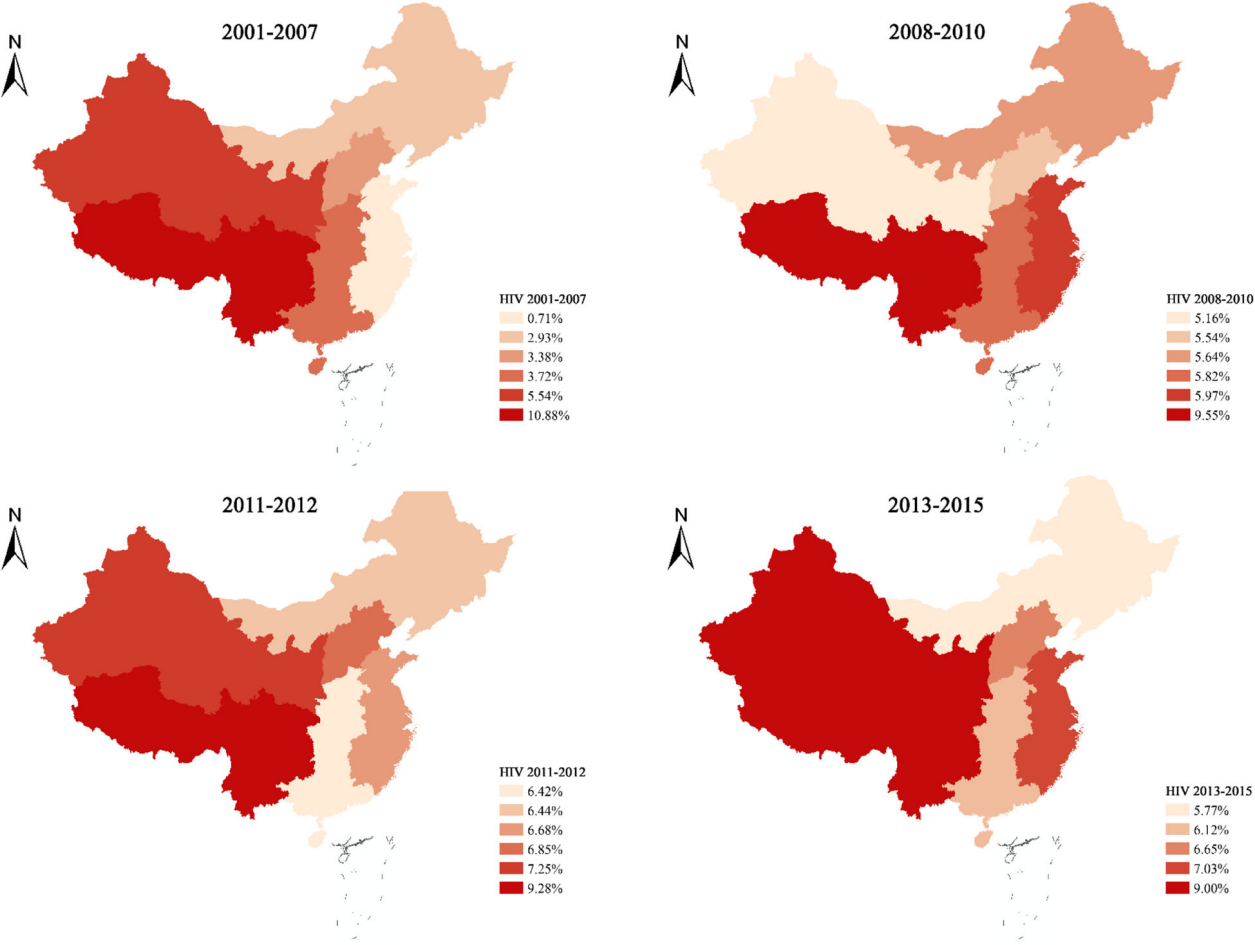
HIV prevalence among MSM in 6 geographical regions. Data presented were based on independent studies from 2001 to 2015

**Fig. 2:**
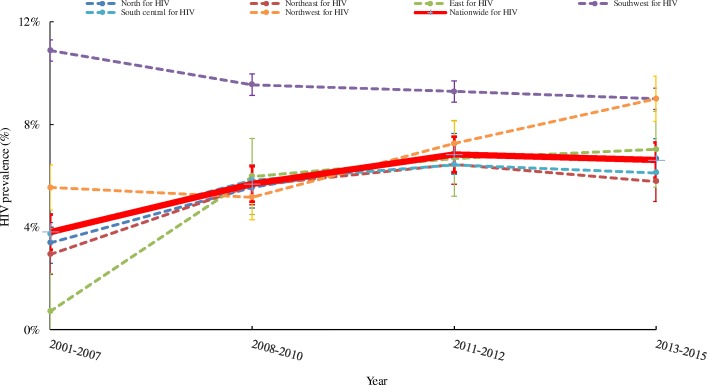
Temporal trends of estimated HIV prevalence nationwide and in 6 geographical regions. The trends were estimated based on data from 2001 to 2015 independent studies

Our data shows a steadily rising trend in HIV prevalence among MSM in China, converging across all regions (Figs. 1 and 2). The pooled national HIV prevalence increased from 3.8% (95% CI: 3.0–4.8%) in 2001–2007 to 6.6% (95% CI: 5.6–7.7%) in 2013–2015. The HIV prevalence in the Southwest region showed a slightly downward trend [10.9% (95% CI: 9.8–12.1%) in 2001–2007, to 9.0% (95% CI: 6.2–12.9%) in 2013–2015). The Northwest (from 5.5, 95% CI: 4.4–7.0% in 2001–2007, to 9.0, 95% CI: 4.9–16.1% in 2013–2015) and East (from 0.7% (95% CI: 0.1–1.9%) in 2001–2007, to 7.0% (95% CI: 5.9–8.3%) in 2013–2015) regions showed increasing HIV prevalence over the years, of which the prevalence surged tenfold in the East during the study period. The trends of HIV prevalence in the North, Northeast, and South-central regions were similar, rising from around 3.0% in 2001–2007 to 7.0% in 2008–2010 and remained more or less stable since then.

**Fig. 3:**
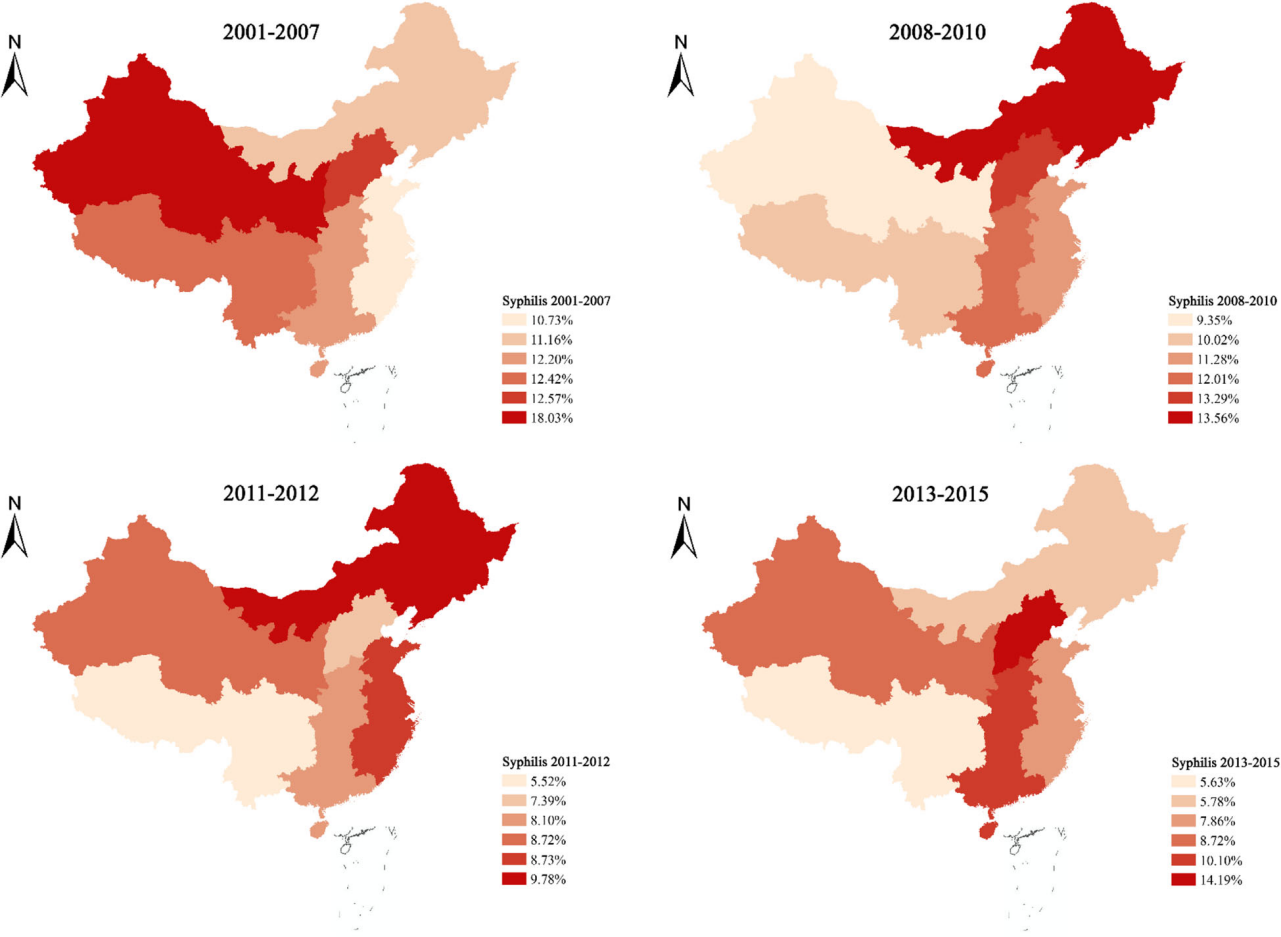
Syphilis prevalence among MSM in 6 geographical regions. Data presented were based on independent studies from 2001 to 2015

The syphilis prevalence in mainland China decreased from 12.3% (95% CI: 10.2–14.9%) in 2001–2007 to 7.1% (95% CI: 5.6–8.9%) in 2013–2015 in a fluctuating pattern (Fig. 3 and Additional file 4). The largest declines in percentage-point were observed in the northwest and southwest regions, decreasing from 18.0% (95% CI: 5.5–45.4%) in 2001–2007 to 8.7% (95% CI: 4.5–16.3%) in 2013–2015 in the northwest and 12.4% (95% CI: 6.5–22.4%) in 2001–2007 to 5.6% (95% CI: 6.3–9.6%) in 2013–2015 in the southwest, respectively. The East, Northeast, and South-central regions had a modest decrease of 2.0–6.0% from 2001 to 2015 (Fig. 3 and Additional file 4). The prevalence of syphilis in the North showed a dramatic changing trend, decreasing from 12.6% (95% CI: 8.7–17.8%) in 2001–2007 to 7.4% in 2011–2012, then rising sharply and peaking at 14.2% (95% CI: 5.9–30.3%) in 2013–2015.

We noted a modest reduction in the rate of condomless sex with male partners P1M (Tables 2 and 3). In the eastern region, this rate decreased from 61.3% in 2008–2010 to 55.0% in 2013–2015. However, in the south-central region, it fluctuated around 75.0% in 2008–2015. Condomless sex rate with female partners P3M decreased moderately during 2008–2015, from 34.0 to 22.7% in the eastern region and from 11.1 to 6.1% in the southwestern region. The rate of condomless sex with casual male partner P3M varied in different regions: in the East, this rate reduced from 66.1% in 2008–2010 to 60.7% in 2013–2015, while in the Southwest, it rose from 43.1 to 55.5% over the same period.

**Table 2:** Prevalence of HIV and syphilis among MSM in 6 geographical regions

Region	Prevalence of HIV (%, 95 CI)				Prevalence of syphilis (%, 95 CI)			
	2001–2007	2008–2010	2011–2012	2013–2015	2001–2007	2008–2010	2011–2012	2013–2015
North	3.38 (2.38–4.79)	5.54 (4.76–6.64)	6.85 (4.56–10.17)	6.65 (3.30–12.95)	12.57 (8.73–17.78)	13.29 (11.26–15.62)	7.39 (6.54–8.34)	14.19 (5.93–30.25)
Northeast	2.93 (1.59–5.33)	5.64 (5.16–6.15)	6.44 (2.75–7.21)	5.77 (3.98–8.30)	11.16 (4.44–25.39)	13.56 (11.88–15.44)	9.78 (7.69–12.36)	5.78 (1.75–17.46)
East	0.71 (0.10–1.85)	5.97 (5.14–6.92)	6.68 (5.73–7.77)	7.03 (5.94–8.31)	10.73 (7.09–15.91)	11.28 (9.98–12.72)	8.73 (7.48–10.15)	7.86 (6.25–9.85)
Southwest	10.88 (9.78–12.10)	9.55 (8.05–11.29)	9.28 (7.81–11.00)	9.00 (6.21–12.86)	12.42 (6.51–22.42)	10.02 (8.81–11.37)	5.52 (4.61–6.60)	5.63 (6.25–9.60)
South central	3.72 (2.51–5.50)	5.82 (5.36–6.33)	6.42 (5.68–7.25)	6.12 (4.95–7.54)	12.20 (8.81–16.64)	12.01 (10.35–13.90)	8.10 (6.88–9.52)	10.10 (5.35–18.25)
Northwest	5.54 (4.36–7.01)	5.16 (4.66–5.70)	7.25 (5.08–10.25)	9.00 (4.85–16.09)	18.03 (5.50–45.43)	9.35 (7.50–11.62)	8.72 (4.47–16.31)	8.72 (4.47–16.31)
Nationwide	3.80 (3.03–4.76)	5.68 (5.14–6.28)	6.83 (6.17–7.55)	6.61 (5.65–7.71)	12.34 (10.16–14.92)	11.00 (9.97–12.12)	7.87 (7.03–8.79)	7.09 (5.59–8.94)

**Table 3:** High-risk behavior rates among MSM in 6 geographical regions^a^

	Condomless sex with male partner P1M (*n*, %)			Condomless sex with female P3M (*n*, %)			Unprotected sex with casual male partner P3M (*n*, %)		
Region	2008–2010	2011–2012	2013–2015	2008–2010	2011–2012	2013–2015	2008–2010	2011–2012	2013–2015
North	14,267 (64.0)	1095 (55.5)	–	–	–	–	241 (45.1)	145 (35.5)	–
Northeast	14,135 (51.6)	–	–	62 (29.1)	183 (34.9)	–	388 (46.0)	401 (64.1)	–
East	21,469 (61.3)	2594 (56.0)	2520 (55.0)	1868 (34.0)	2330 (35.6)	581 (22.7)	897 (66.1)	600 (67.9)	1351 (60.7)
Southwest	21,614 (63.8)	2094 (53.5)	–	66 (11.1)	80 (10.9)	12 (6.1)	96 (43.1)	1100 (68.7)	227 (55.5)
South central	49,365 (74.6)	10,110 (70.7)	1555 (74.4)	–	320 (29.1)	–	370 (52.6)	149 (56.5)	–
Northwest	29,593 (71.2)	1558 (64.7)	–	–	–	–	196 (81.0)	139 (54.8)	–

^a^There was no study reported behavioral information in 2001–2007

## Discussion

China has been experiencing various changing trends of HIV and syphilis prevalence over time in different regions. Our review extends the literature by evaluating the spatial patterns of HIV, syphilis and condomless sex, by assessing the temporal trend of these measures, and exploring the association between HIV prevalence, syphilis prevalence and condomless sex rate among Chinese MSM. We found an upward trend of HIV prevalence but a declined syphilis prevalence among Chinese MSM, while regional and temporal disparity of these measures were also found between 2001 and 2015.

We found the increase of HIV prevalence mostly occurred before 2010, and it remained stable since then except in the Northwestern region. The relatively stable HIV prevalence between 2011 and 2015 was contrasted with from the trend observed from China’s national AIDS sentinel surveillance system [7, 19]. While the global prevalence among MSM is still increasing, [1, 27] the relative stable HIV prevalence among Chinese MSM, except for those in the Northwest, is encouraging. There are a few reasons for the stabilization in HIV prevalence among Chinese MSM since 2011, following the preceding rise. First, this may be due to the scaling-up of HIV testing services and broader coverage of treatment services targeting MSM since 2008 [28]. Second, the roll-out of HIV prevention programs targeting MSM population in the country, including educational campaigns, promotion of safer sex and condom use, control of drug use, has contributed to the control of HIV spread among the group [29]. However, all of these results were based on prevalence data, and mathematical modelling are needed to estimate the underlying incidence and reaffirm this trend.

We also found that HIV prevalence in the East and the Northwest increased most significantly between 2001 and 2012. For example, the HIV prevalence in Eastern China increased about 10 times, even though the trend turned stable in the later time periods. This change may be due to a high incidence rate caused by common condomless anal sex facilitated by the fast expansion of cruising venues (e.g. pubs, spas, saunas etc.) [30], increased use of dating apps, [31] and the migration of Chinese MSM in the eastern region [32]. Nevertheless, HIV prevalence consistently increased in Northwest China and reached the highest level at 9% since 2008. We recommend that more attention and health resources should be directed to this region, and studies are needed to examine the reasons for this continuous increase.

The overall trend of syphilis prevalence has been decreasing with fluctuation across the country. The Northwest was a main contributing region (decreased by more than 50%). The changes in syphilis prevalence in the Northwest, East, and Northeast tend to be in parallel to increased regional HIV prevalence during the examined timeframe. Considering that syphilis is a completely curable disease but not HIV, main factors could be an expanded coverage of early screening and treatment services and other interventions aiming at behavioral changes among MSM [33, 34]. After 2008, all regions had reduced or stable syphilis prevalence except for the North which started to climb up rapidly since 2011. Reasons for such a sharp rise are underexplored and warrant further research. Relevant authorities should be attentive to the resurgence of syphilis, especially in northern China.

The patterns of condomless sex with male partners varied with regions, with the North, the East and the Northwest showing decreases while other regions revealing either increasing or unchanging trends. The rates of condomless sex remained at consistently high levels in different regions which are similar to those in the United States [35] and United Kingdom [36]. Previous efforts may have some positive effects on bringing down condomless sex prevalence in some regions. However, considering that behavioral change is a challenging complex process, efforts should be expanded further to control sexual risky behaviors nationwide.

This study has some limitations. First, we did not evaluate publication bias, significant results are more likely to be published and the prevalence rates in this study might magnify the results. Second, the scaled-up big-city-level national sentinel surveillance might bias the geographical characteristics of HIV prevalence, which might disproportionately underestimate the HIV epidemic in some less-developed regions over time. Third, the same data may have been reported in more than one out of the included 272 articles, and we could not identify and exclude all the overlapped data because some province-level studies did not specify data of different cities while data of some city-level studies we selected might be duplicated. For example, Qin et al. reported the national HIV prevalence among Chinese based on the sentinel surveillance data, and these data may have also been used in some local studies [19].

## Conclusions

In conclusion, our findings suggested that the HIV prevalence has shown an increasing trend nationwide before 2010. However, it appears to stabilize in most regions afterward except in the Northwest, indicating more attention should be directed into this area. The estimated decline in syphilis correlated with national syphilis control efforts. Nevertheless, it should be noted that northern China has seen a sharp increase in recent years. This warrants more resources to prevent the resurgence of syphilis in this area. In addition, given the consistent high condomless rate among Chinese MSM, policies and strategies to improve safe sex are needed.

## Additional files

Additional file 1: Flow chart showing the meta-analysis studies selection. The flow chart presents the process of studies searching, screening, and assessment. (PDF 113 kb)

Additional file 2: Dataset listing extracted data of 272 articles included. The dataset includes periodic and demographic information, the crude HIV and syphilis prevalence rate, and history of condom use with male partner, female, and unprotected sex with casual male partner. (XLSX 37 kb)

Additional file 3: Meta-analyses of HIV and syphilis prevalence among MSM, stratified by 6 geographical regions. The meta-analyses present prevalence rates in forest plots, fixed effect model and random effects model. (PDF 1622 kb)

Additional file 4: Temporal trends of estimated syphilis prevalence nationwide and in 6 geographical regions. The trends were estimated based on data from 2001-2015 independent studies. (PDF 279 kb)

## Abbreviation

AIDS: Acquired Immune Deficiency Syndrome
HIV: Human Immunodeficiency Virus
MSM: Men Who Have Sex with Men
P1M: In the Past One Month
P3M: In the Past Three Months
STIs: Sexually Transmitted Infections
