# Supplementary figures and images for: A spatiotemporal meta-analysis of HIV/syphilis epidemic among men who have sex with men living in mainland China
Source: BMC Infect Dis. 2018 Dec 12;18:652. doi: 10.1186/s12879-018-3532-8 (PMC6292157; doi:10.1186/s12879-018-3532-8)

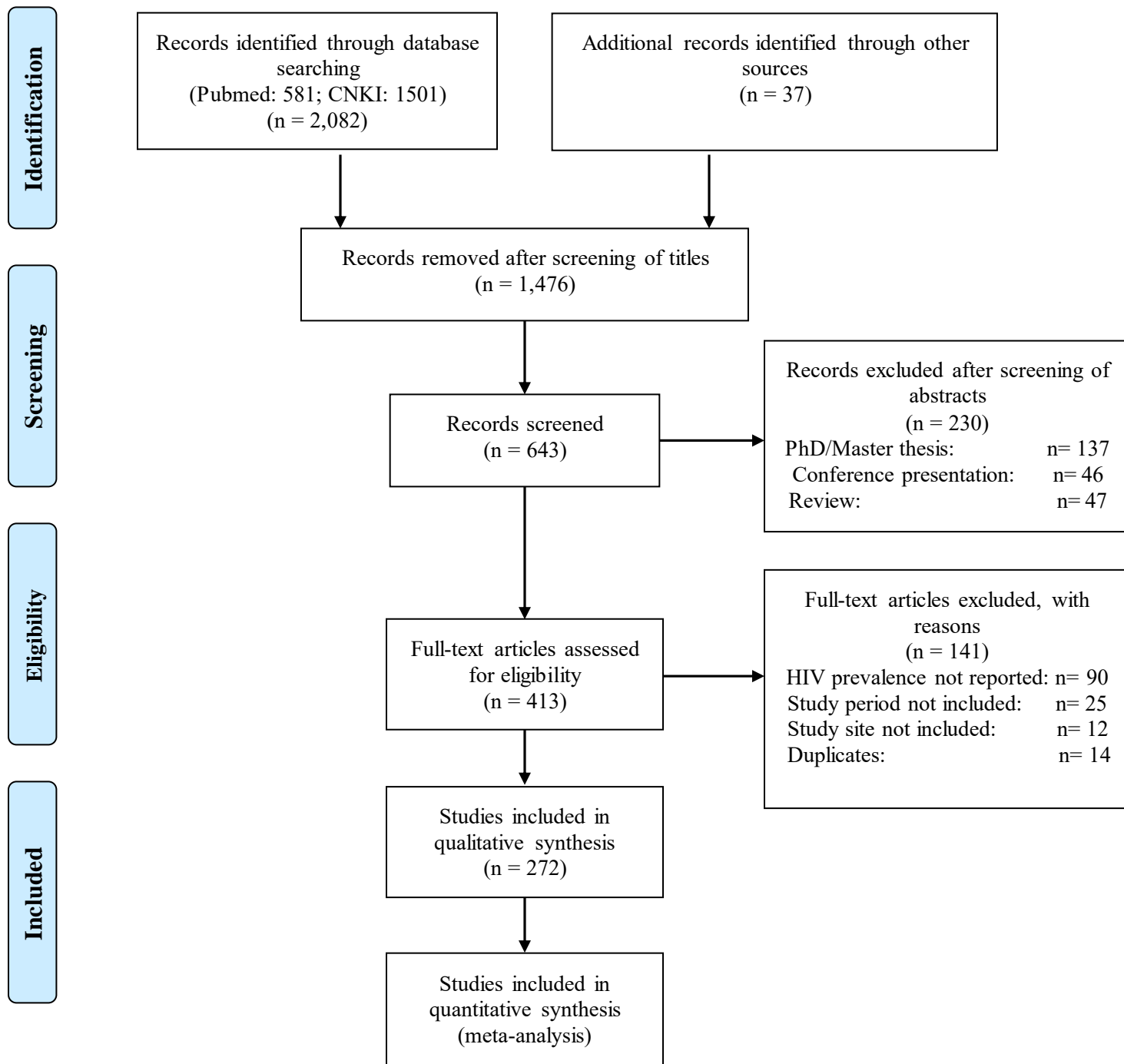

Supplement: Supplementary file 1 — Flow chart showing the meta-analysis studies selection. The flow chart presents the process of studies searching, screening, and assessment. (PDF 113 kb) [file 12879_2018_3532_MOESM1_ESM.pdf]

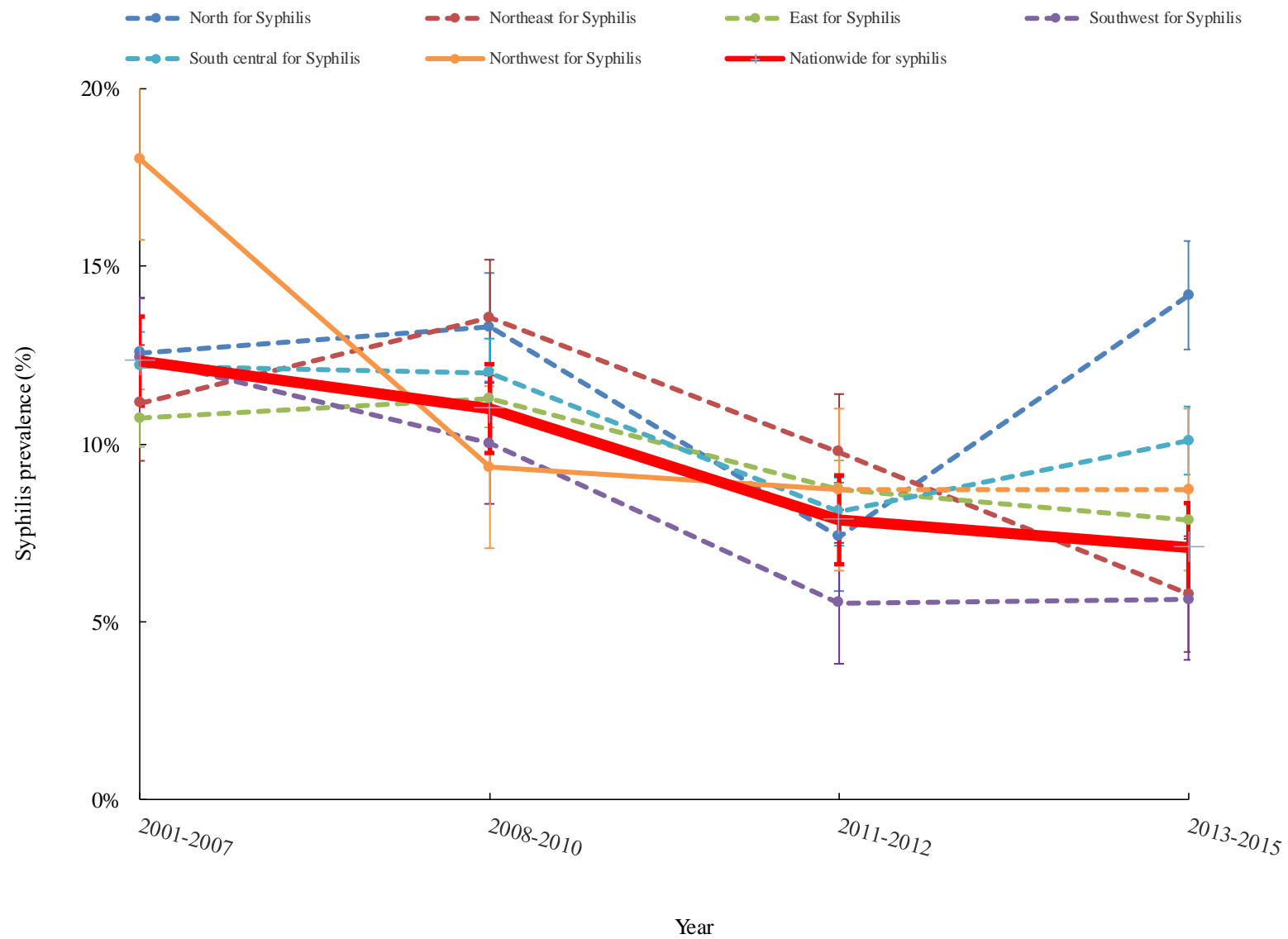

Supplement: Supplementary file 4 — Temporal trends of estimated syphilis prevalence nationwide and in 6 geographical regions. The trends were estimated based on data from 2001-2015 independent studies. (PDF 279 kb) [file 12879_2018_3532_MOESM4_ESM.pdf]
