# Supplementary material for: A spatiotemporal meta-analysis of HIV/syphilis epidemic among men who have sex with men living in mainland China
Source: BMC Infect Dis. 2018 Dec 12;18:652. doi: 10.1186/s12879-018-3532-8 (PMC6292157; doi:10.1186/s12879-018-3532-8)
Supplement: Supplementary file 3 — Meta-analyses of HIV and syphilis prevalence among MSM, stratified by 6 geographical regions. The meta-analyses present prevalence rates in forest plots, fixed effect model and random effects model. (PDF 1622 kb) [file 12879_2018_3532_MOESM3_ESM.pdf]

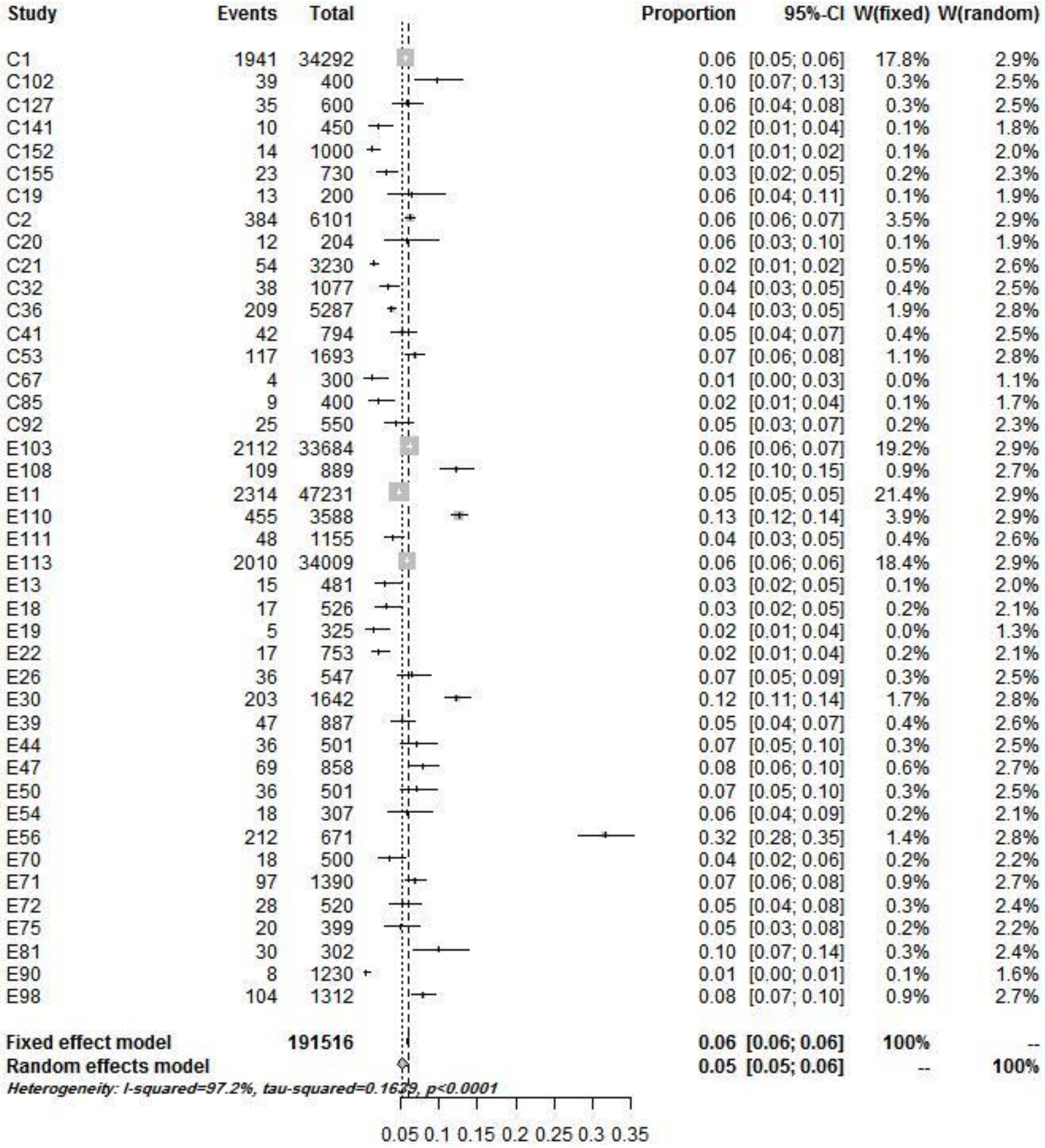

Meta-analysis of HIV prevalence in among MSM in North China

Study	Events	Total	Proportion	95%-CI	W(fixed)	W(random)
C1	1941	34292	0.06	[0.05; 0.06]	9.5%	3.8%
C10	100	2074	0.05	[0.04; 0.06]	0.5%	3.2%
C109	34	492	0.07	[0.05; 0.10]	0.2%	2.4%
C110	24	400	0.06	[0.04; 0.09]	0.1%	2.1%
C115	6	407	0.01	[0.01; 0.03]	0.0%	0.9%
C116	30	806	0.04	[0.03; 0.05]	0.2%	2.3%
C126	31	400	0.08	[0.05; 0.11]	0.1%	2.3%
C129	20	604	0.03	[0.02; 0.05]	0.1%	1.9%
C134	7	324	0.02	[0.01; 0.04]	0.0%	1.0%
C139	11	401	0.03	[0.01; 0.05]	0.1%	1.4%
C140	46	802	0.06	[0.04; 0.08]	0.2%	2.7%
C15	18	402	0.04	[0.03; 0.07]	0.1%	1.8%
C154	12	475	0.03	[0.01; 0.04]	0.1%	1.5%
C172	70	731	0.10	[0.08; 0.12]	0.3%	2.9%
C173	63	603	0.10	[0.08; 0.13]	0.3%	2.9%
C180	19	3200	0.01	[0.00; 0.01]	0.1%	1.9%
C2	384	6101	0.06	[0.06; 0.07]	1.9%	3.6%
C27	17	401	0.04	[0.02; 0.07]	0.1%	1.8%
C49	33	1172	0.03	[0.02; 0.04]	0.2%	2.4%
C53	117	1693	0.07	[0.06; 0.08]	0.6%	3.2%
C70	12	475	0.03	[0.01; 0.04]	0.1%	1.5%
C86	58	1258	0.05	[0.04; 0.06]	0.3%	2.8%
E10	4	401	0.01	[0.00; 0.03]	0.0%	0.7%
E103	2112	33684	0.06	[0.06; 0.07]	10.3%	3.8%
E11	2314	47231	0.05	[0.05; 0.05]	11.4%	3.8%
E112	228	2958	0.08	[0.07; 0.09]	1.1%	3.5%
E113	11454	162576	0.07	[0.07; 0.07]	55.4%	3.8%
E15	31	1353	0.02	[0.02; 0.03]	0.2%	2.4%
E16	13	229	0.06	[0.03; 0.10]	0.1%	1.5%
E27	354	3834	0.09	[0.08; 0.10]	1.7%	3.6%
E45	31	626	0.05	[0.03; 0.07]	0.2%	2.3%
E47	69	858	0.08	[0.06; 0.10]	0.3%	2.9%
E55	116	1765	0.07	[0.05; 0.08]	0.6%	3.2%
E71	97	1390	0.07	[0.06; 0.08]	0.5%	3.2%
E74	44	463	0.10	[0.07; 0.13]	0.2%	2.6%
E83	2	215	0.01	[0.00; 0.03]	0.0%	0.4%
E84	13	218	0.06	[0.03; 0.10]	0.1%	1.5%
E86	226	2936	0.08	[0.07; 0.09]	1.1%	3.5%
E95	228	2958	0.08	[0.07; 0.09]	1.1%	3.5%
E99	195	2618	0.07	[0.06; 0.09]	0.9%	3.4%
**Fixed effect model**		**323826**	**0.06**	**[0.06; 0.07]**	**100%**	--
**Random effects model**			**0.05**	**[0.05; 0.06]**	--	**100%**

Heterogeneity: I-squared=94.6%, tau-squared=0.0543, p<0.0001

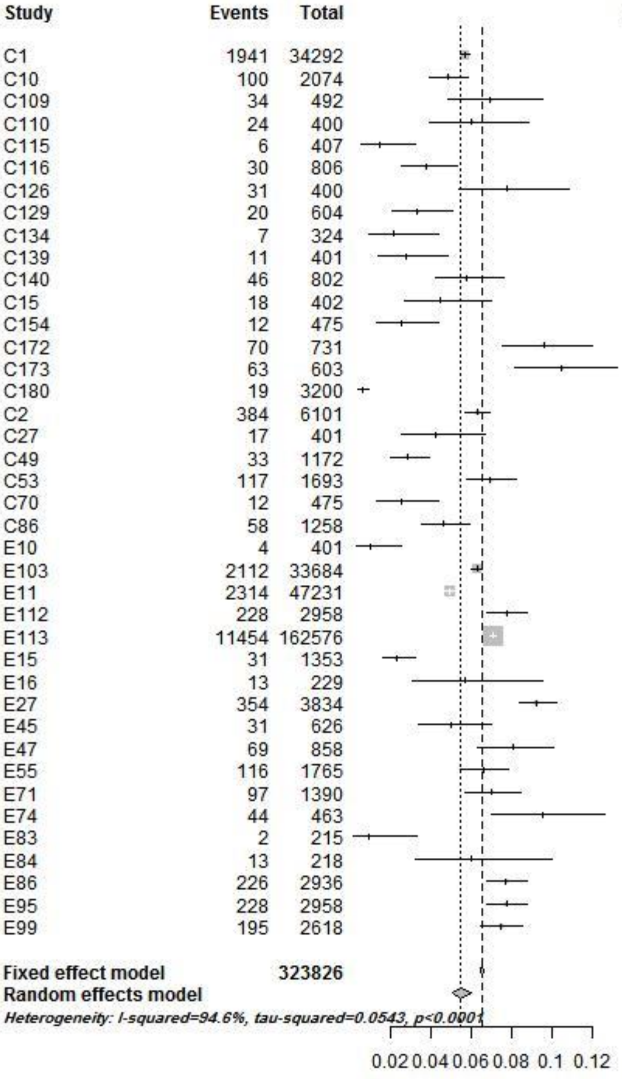

Meta-analysis of HIV prevalence in among MSM in Northeast China

Study	Events	Total	Proportion	95%-CI	W(fixed)	W(random)
C1	1941	34292	0.06	[0.05; 0.06]	9.1%	2.1%
C108	4	228	0.02	[0.00; 0.04]	0.0%	0.6%
C111	18	918	0.02	[0.01; 0.03]	0.1%	1.3%
C112	49	1542	0.03	[0.02; 0.04]	0.2%	1.7%
C113	5	260	0.02	[0.01; 0.04]	0.0%	0.7%
C114	11	400	0.03	[0.01; 0.05]	0.1%	1.0%
C12	2	158	0.01	[0.00; 0.04]	0.0%	0.3%
C122	22	520	0.04	[0.03; 0.06]	0.1%	1.4%
C123	0	55	0.00	[0.00; 0.06]	0.0%	0.1%
C133	14	490	0.03	[0.02; 0.05]	0.1%	1.2%
C136	14	401	0.03	[0.02; 0.06]	0.1%	1.2%
C137	71	750	0.09	[0.07; 0.12]	0.3%	1.8%
C14	9	306	0.03	[0.01; 0.06]	0.0%	0.9%
C142	7	452	0.02	[0.01; 0.03]	0.0%	0.8%
C145	36	404	0.09	[0.06; 0.12]	0.2%	1.6%
C146	8	126	0.06	[0.03; 0.12]	0.0%	0.9%
C148	11	155	0.07	[0.04; 0.12]	0.1%	1.0%
C149	114	3164	0.04	[0.03; 0.04]	0.5%	1.9%
C150	292	2892	0.10	[0.09; 0.11]	1.3%	2.0%
C153	8	165	0.05	[0.02; 0.09]	0.0%	0.9%
C156	90	1200	0.08	[0.06; 0.09]	0.4%	1.9%
C157	25	403	0.06	[0.04; 0.09]	0.1%	1.4%
C158	53	1225	0.04	[0.03; 0.06]	0.3%	1.7%
C166	15	208	0.07	[0.04; 0.12]	0.1%	1.2%
C174	50	1093	0.05	[0.03; 0.06]	0.2%	1.7%
C176	25	329	0.08	[0.05; 0.11]	0.1%	1.4%
C182	25	403	0.06	[0.04; 0.09]	0.1%	1.4%
C183	91	1752	0.05	[0.04; 0.06]	0.4%	1.9%
C2	384	6101	0.06	[0.06; 0.07]	1.8%	2.0%
C22	60	790	0.08	[0.06; 0.10]	0.3%	1.8%
C33	4	61	0.07	[0.02; 0.16]	0.0%	0.5%
C35	20	421	0.05	[0.03; 0.07]	0.1%	1.3%
C38	42	436	0.10	[0.07; 0.13]	0.2%	1.6%
C47	12	474	0.03	[0.01; 0.04]	0.1%	1.1%
C48	7	274	0.03	[0.01; 0.05]	0.0%	0.8%
C6	0	144	0.00	[0.00; 0.03]	0.0%	0.1%
C71	39	2680	0.01	[0.01; 0.02]	0.2%	1.6%
C72	25	830	0.03	[0.02; 0.04]	0.1%	1.5%
C73	15	265	0.06	[0.03; 0.09]	0.1%	1.2%
C74	58	506	0.11	[0.09; 0.15]	0.3%	1.7%
C76	8	687	0.01	[0.01; 0.02]	0.0%	0.9%
C78	80	597	0.13	[0.11; 0.16]	0.3%	1.8%
C8	25	421	0.06	[0.04; 0.09]	0.1%	1.4%
C83	15	403	0.04	[0.02; 0.06]	0.1%	1.2%
C90	12	169	0.07	[0.04; 0.12]	0.1%	1.1%
C91	18	294	0.06	[0.04; 0.10]	0.1%	1.3%
E100	54	589	0.09	[0.07; 0.12]	0.2%	1.7%
E101	57	535	0.11	[0.08; 0.14]	0.3%	1.7%
E102	48	413	0.12	[0.09; 0.15]	0.2%	1.7%
E103	2112	33684	0.06	[0.06; 0.07]	9.8%	2.1%
E104	98	424	0.23	[0.19; 0.27]	0.4%	1.8%
E106	23	259	0.09	[0.06; 0.13]	0.1%	1.4%
E11	2314	47231	0.05	[0.05; 0.05]	10.9%	2.1%
E112	228	2958	0.08	[0.07; 0.09]	1.0%	2.0%
E113	11454	162576	0.07	[0.07; 0.07]	52.8%	2.1%
E115	73	587	0.12	[0.10; 0.15]	0.3%	1.8%
E17	7	475	0.01	[0.01; 0.03]	0.0%	0.8%
E20	3	428	0.01	[0.00; 0.02]	0.0%	0.4%
E25	46	352	0.13	[0.10; 0.17]	0.2%	1.7%
E27	354	3834	0.09	[0.08; 0.10]	1.6%	2.0%
E34	20	430	0.05	[0.03; 0.07]	0.1%	1.3%
E35	0	144	0.00	[0.00; 0.03]	0.0%	0.1%
E36	20	280	0.07	[0.04; 0.11]	0.1%	1.3%
E43	20	1230	0.02	[0.01; 0.03]	0.1%	1.4%
E58	19	416	0.05	[0.03; 0.07]	0.1%	1.3%
E59	20	430	0.05	[0.03; 0.07]	0.1%	1.3%
E64	75	2996	0.03	[0.02; 0.03]	0.4%	1.8%
E82	19	397	0.05	[0.03; 0.07]	0.1%	1.3%
E86	226	2936	0.08	[0.07; 0.09]	1.0%	2.0%
E88	70	620	0.11	[0.09; 0.14]	0.3%	1.8%
E95	228	2958	0.08	[0.07; 0.09]	1.0%	2.0%
E99	195	2618	0.07	[0.06; 0.09]	0.9%	2.0%
**Fixed effect model**		**339644**	**0.07**	**[0.06; 0.07]**	**100%**	--
**Random effects model**			**0.06**	**[0.05; 0.06]**	--	**100%**

Heterogeneity: I-squared=94.7%, tau-squared=0.0909, p<0.0001

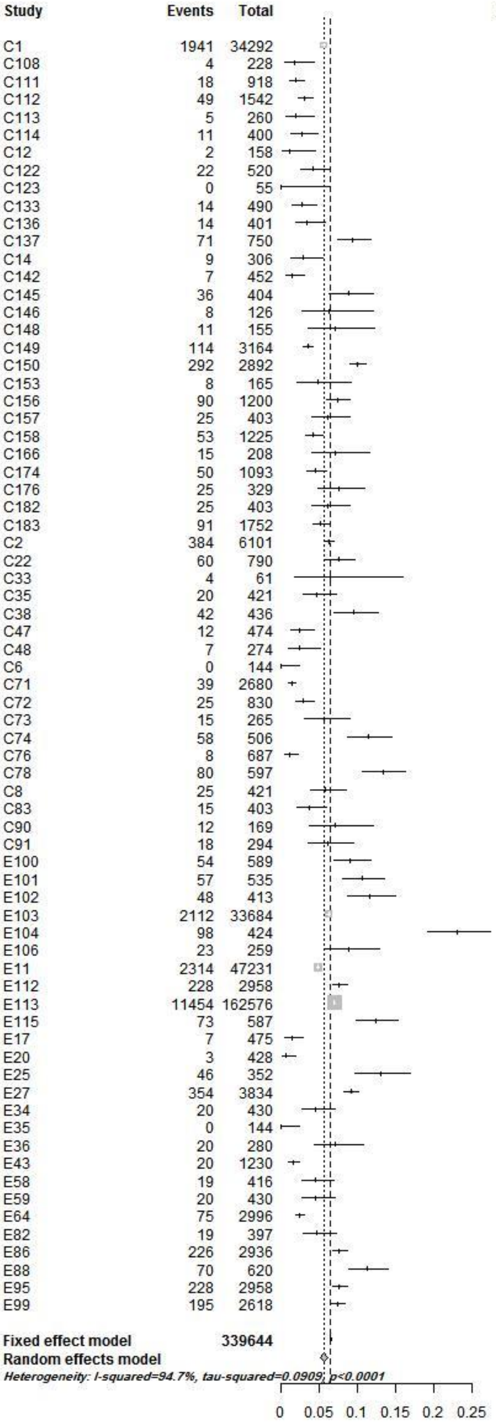

Meta-analysis of HIV prevalence in among MSM in East China

Study	Events	Total	Proportion	95%-CI	W(fixed)	W(random)
C1	1941	34292	0.06	[0.05; 0.06]	8.7%	2.6%
C11	41	251	0.16	[0.12; 0.22]	0.2%	2.2%
C121	13	423	0.03	[0.02; 0.05]	0.1%	1.7%
C125	1	19	0.05	[0.00; 0.26]	0.0%	0.3%
C130	40	1038	0.04	[0.03; 0.05]	0.2%	2.2%
C151	246	3611	0.07	[0.06; 0.08]	1.1%	2.6%
C17	332	2889	0.11	[0.10; 0.13]	1.4%	2.6%
C175	184	922	0.20	[0.17; 0.23]	0.7%	2.5%
C2	384	6101	0.06	[0.06; 0.07]	1.7%	2.6%
C24	89	366	0.24	[0.20; 0.29]	0.3%	2.4%
C40	75	341	0.22	[0.18; 0.27]	0.3%	2.3%
C43	45	458	0.10	[0.07; 0.13]	0.2%	2.2%
C53	117	1693	0.07	[0.06; 0.08]	0.5%	2.5%
C63	17	405	0.04	[0.02; 0.07]	0.1%	1.8%
C64	44	400	0.11	[0.08; 0.14]	0.2%	2.2%
C65	61	820	0.07	[0.06; 0.09]	0.3%	2.3%
C7	22	911	0.02	[0.02; 0.04]	0.1%	2.0%
C81	224	2527	0.09	[0.08; 0.10]	1.0%	2.5%
C89	123	743	0.17	[0.14; 0.19]	0.5%	2.5%
C93	70	469	0.15	[0.12; 0.18]	0.3%	2.3%
C98	11	109	0.10	[0.05; 0.17]	0.0%	1.5%
C99	193	1045	0.18	[0.16; 0.21]	0.7%	2.5%
E103	2112	33684	0.06	[0.06; 0.07]	9.4%	2.6%
E107	73	391	0.19	[0.15; 0.23]	0.3%	2.3%
E108	109	889	0.12	[0.10; 0.15]	0.5%	2.4%
E109	49	1195	0.04	[0.03; 0.05]	0.2%	2.3%
E11	2314	47231	0.05	[0.05; 0.05]	10.4%	2.6%
E112	228	2958	0.08	[0.07; 0.09]	1.0%	2.5%
E113	11454	162576	0.07	[0.07; 0.07]	50.5%	2.6%
E27	354	3834	0.09	[0.08; 0.10]	1.5%	2.6%
E28	59	510	0.12	[0.09; 0.15]	0.2%	2.3%
E3	104	1000	0.10	[0.09; 0.12]	0.4%	2.4%
E33	76	568	0.13	[0.11; 0.16]	0.3%	2.4%
E47	69	858	0.08	[0.06; 0.10]	0.3%	2.4%
E53	49	513	0.10	[0.07; 0.12]	0.2%	2.3%
E71	97	1390	0.07	[0.06; 0.08]	0.4%	2.4%
E78	79	503	0.16	[0.13; 0.19]	0.3%	2.4%
E8	73	561	0.13	[0.10; 0.16]	0.3%	2.4%
E80	182	1692	0.11	[0.09; 0.12]	0.8%	2.5%
E85	375	2422	0.15	[0.14; 0.17]	1.5%	2.6%
E86	226	2936	0.08	[0.07; 0.09]	1.0%	2.5%
E95	228	2958	0.08	[0.07; 0.09]	1.0%	2.5%
E99	195	2618	0.07	[0.06; 0.09]	0.9%	2.5%
**Fixed effect model**		**331120**	**0.07**	**[0.07; 0.07]**	**100%**	--
**Random effects model**			**0.09**	**[0.08; 0.10]**	--	**100%**

Heterogeneity: I-squared=97.9%, tau-squared=0.1323, p<0.0001

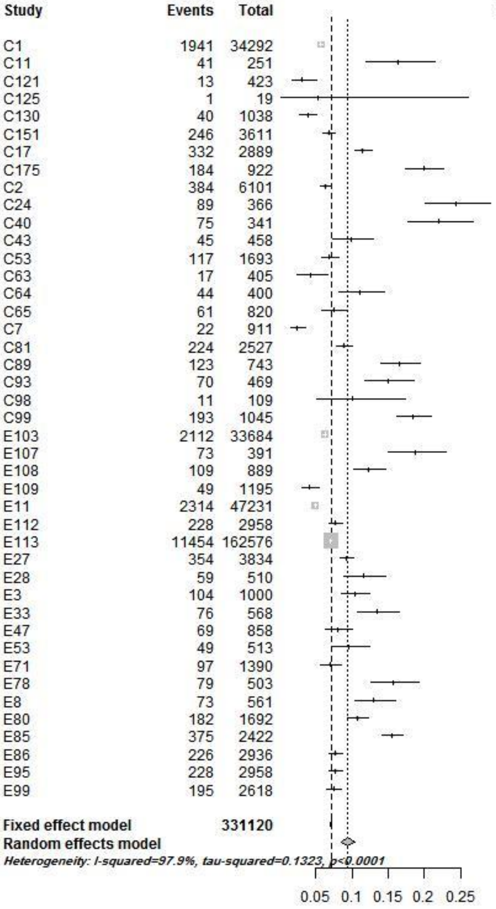

Meta-analysis of HIV prevalence in among MSM in Southwest China

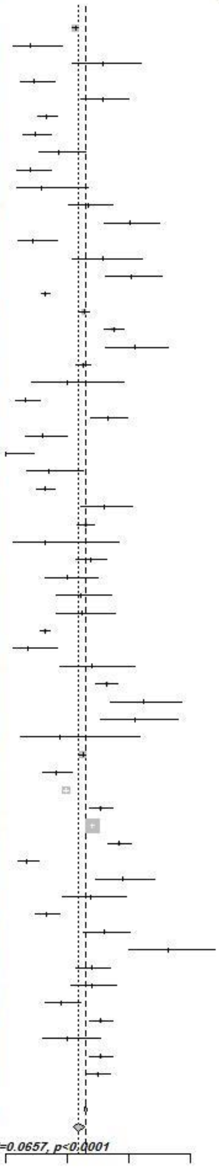

Study	Events	Total	Proportion	95%-CI	W(fixed)	W(random)
C1	1941	34292	0.06	[0.05; 0.06]	8.3%	2.4%
C100	5	248	0.02	[0.01; 0.05]	0.0%	0.6%
C101	31	393	0.08	[0.05; 0.11]	0.1%	1.6%
C103	13	555	0.02	[0.01; 0.04]	0.1%	1.1%
C104	61	774	0.08	[0.06; 0.10]	0.3%	1.9%
C105	73	2187	0.03	[0.03; 0.04]	0.3%	2.0%
C106	18	752	0.02	[0.01; 0.04]	0.1%	1.3%
C107	22	507	0.04	[0.03; 0.06]	0.1%	1.4%
C132	9	452	0.02	[0.01; 0.04]	0.0%	0.9%
C135	5	170	0.03	[0.01; 0.07]	0.0%	0.6%
C138	54	800	0.07	[0.05; 0.09]	0.2%	1.8%
C143	72	716	0.10	[0.08; 0.12]	0.3%	1.9%
C144	9	404	0.02	[0.01; 0.04]	0.0%	0.9%
C159	31	392	0.08	[0.05; 0.11]	0.1%	1.6%
C161	72	707	0.10	[0.08; 0.13]	0.3%	1.9%
C167	301	9251	0.03	[0.03; 0.04]	1.3%	2.3%
C168	910	14268	0.06	[0.06; 0.07]	3.9%	2.4%
C171	429	4904	0.09	[0.08; 0.10]	1.8%	2.3%
C173	63	603	0.10	[0.08; 0.13]	0.3%	1.9%
C2	384	6101	0.06	[0.06; 0.07]	1.6%	2.3%
C28	8	161	0.05	[0.02; 0.10]	0.0%	0.8%
C3	13	790	0.02	[0.01; 0.03]	0.1%	1.1%
C30	119	1432	0.08	[0.07; 0.10]	0.5%	2.1%
C31	14	462	0.03	[0.02; 0.05]	0.1%	1.1%
C4	0	155	0.00	[0.00; 0.02]	0.0%	0.1%
C42	10	287	0.03	[0.02; 0.06]	0.0%	0.9%
C45	74	2296	0.03	[0.03; 0.04]	0.3%	2.0%
C46	54	675	0.08	[0.06; 0.10]	0.2%	1.8%
C5	338	5232	0.06	[0.06; 0.07]	1.4%	2.3%
C51	3	92	0.03	[0.01; 0.09]	0.0%	0.4%
C53	117	1693	0.07	[0.06; 0.08]	0.5%	2.1%
C56	23	456	0.05	[0.03; 0.07]	0.1%	1.4%
C58	30	492	0.06	[0.04; 0.09]	0.1%	1.6%
C60	27	436	0.06	[0.04; 0.09]	0.1%	1.5%
C69	228	7123	0.03	[0.03; 0.04]	1.0%	2.2%
C75	5	273	0.02	[0.01; 0.04]	0.0%	0.6%
C77	21	300	0.07	[0.04; 0.11]	0.1%	1.3%
C79	281	3445	0.08	[0.07; 0.09]	1.2%	2.3%
C84	53	476	0.11	[0.08; 0.14]	0.2%	1.8%
C9	42	400	0.10	[0.08; 0.14]	0.2%	1.7%
C94	4	91	0.04	[0.01; 0.11]	0.0%	0.5%
E103	2112	33684	0.06	[0.06; 0.07]	9.0%	2.4%
E109	49	1195	0.04	[0.03; 0.05]	0.2%	1.8%
E11	2314	47231	0.05	[0.05; 0.05]	10.0%	2.4%
E112	228	2958	0.08	[0.07; 0.09]	1.0%	2.2%
E113	11454	162576	0.07	[0.07; 0.07]	48.3%	2.4%
E27	354	3834	0.09	[0.08; 0.10]	1.5%	2.3%
E31	20	1146	0.02	[0.01; 0.03]	0.1%	1.4%
E38	59	621	0.10	[0.07; 0.12]	0.2%	1.9%
E41	28	408	0.07	[0.05; 0.10]	0.1%	1.5%
E42	46	1376	0.03	[0.02; 0.04]	0.2%	1.8%
E47	69	858	0.08	[0.06; 0.10]	0.3%	1.9%
E65	51	388	0.13	[0.10; 0.17]	0.2%	1.8%
E71	97	1390	0.07	[0.06; 0.08]	0.4%	2.1%
E73	56	801	0.07	[0.05; 0.09]	0.2%	1.9%
E77	38	850	0.04	[0.03; 0.06]	0.2%	1.7%
E86	226	2936	0.08	[0.07; 0.09]	0.9%	2.2%
E9	19	379	0.05	[0.03; 0.08]	0.1%	1.3%
E95	228	2958	0.08	[0.07; 0.09]	1.0%	2.2%
E99	195	2618	0.07	[0.06; 0.09]	0.8%	2.2%
**Fixed effect model**		**373450**	**0.06**	**[0.06; 0.07]**	**100%**	--
**Random effects model**			**0.06**	**[0.05; 0.06]**	--	**100%**

Heterogeneity: I-squared=94.8%, tau-squared=0.0657, p<0.0001

Meta-analysis of HIV prevalence in among MSM in Southcentral China

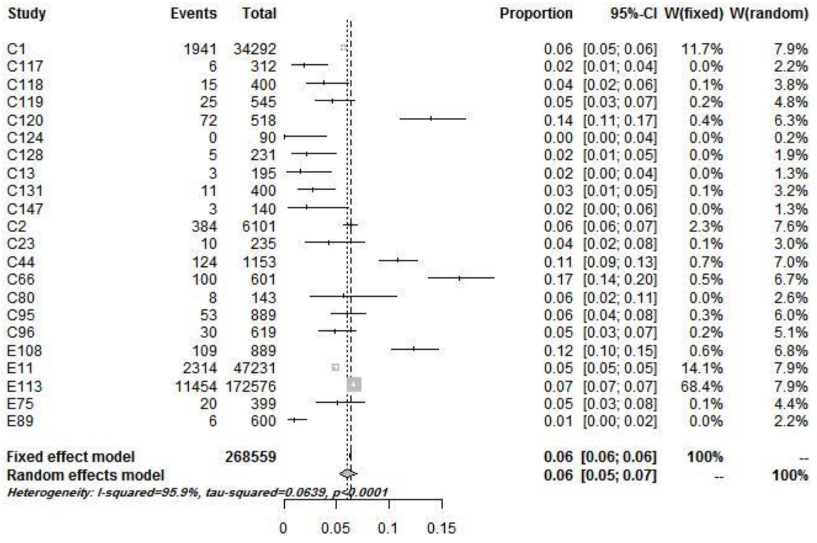

Meta-analysis of HIV prevalence in among MSM in Northwest China

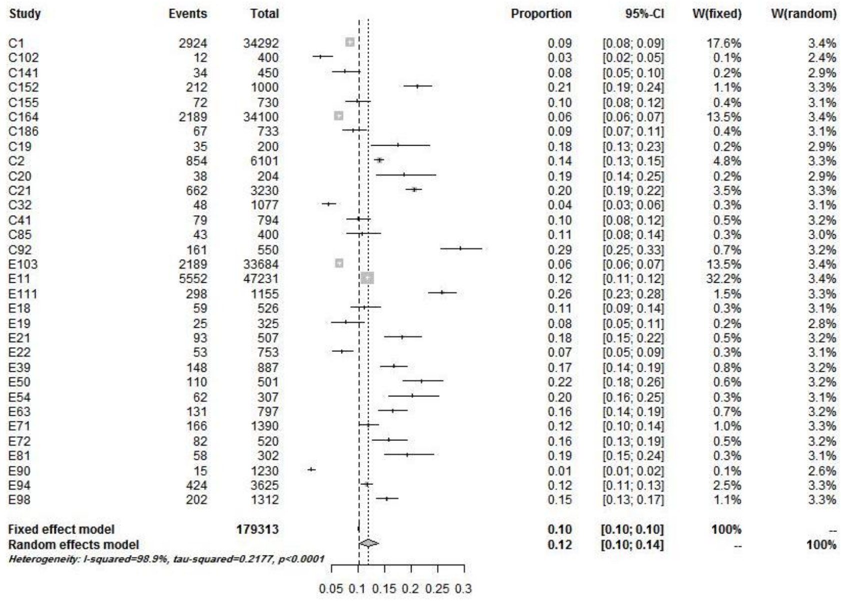

Meta-analysis of syphilis prevalence in among MSM in North China

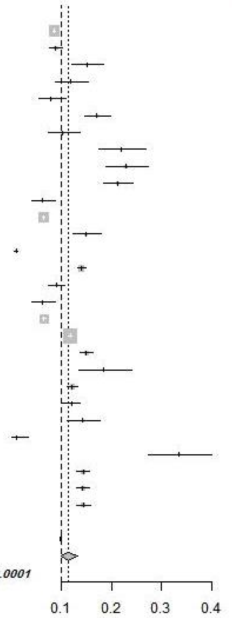

Study	Events	Total	Proportion	95%-CI	W(fixed)	W(random)
C1	2924	34292	0.09	[0.08; 0.09]	17.3%	3.8%
C10	184	2074	0.09	[0.08; 0.10]	1.1%	3.7%
C109	74	492	0.15	[0.12; 0.19]	0.4%	3.5%
C110	47	400	0.12	[0.09; 0.15]	0.3%	3.3%
C115	32	407	0.08	[0.05; 0.11]	0.2%	3.2%
C116	138	806	0.17	[0.15; 0.20]	0.7%	3.6%
C126	41	400	0.10	[0.07; 0.14]	0.2%	3.3%
C134	71	324	0.22	[0.18; 0.27]	0.4%	3.4%
C139	92	401	0.23	[0.19; 0.27]	0.5%	3.5%
C140	171	802	0.21	[0.19; 0.24]	0.9%	3.6%
C154	29	475	0.06	[0.04; 0.09]	0.2%	3.1%
C164	2189	34100	0.06	[0.06; 0.07]	13.2%	3.8%
C173	90	603	0.15	[0.12; 0.18]	0.5%	3.5%
C180	29	3200	0.01	[0.01; 0.01]	0.2%	3.2%
C2	854	6101	0.14	[0.13; 0.15]	4.7%	3.8%
C49	105	1172	0.09	[0.07; 0.11]	0.6%	3.6%
C70	29	475	0.06	[0.04; 0.09]	0.2%	3.1%
E103	2189	33684	0.06	[0.06; 0.07]	13.2%	3.8%
E11	5552	47231	0.12	[0.11; 0.12]	31.6%	3.8%
E112	442	2958	0.15	[0.14; 0.16]	2.4%	3.7%
E16	42	229	0.18	[0.14; 0.24]	0.2%	3.2%
E27	466	3834	0.12	[0.11; 0.13]	2.6%	3.7%
E71	166	1390	0.12	[0.10; 0.14]	0.9%	3.7%
E74	66	463	0.14	[0.11; 0.18]	0.4%	3.4%
E83	2	215	0.01	[0.00; 0.03]	0.0%	1.0%
E84	73	218	0.33	[0.27; 0.40]	0.3%	3.4%
E86	420	2936	0.14	[0.13; 0.16]	2.3%	3.7%
E95	422	2958	0.14	[0.13; 0.16]	2.3%	3.7%
E99	375	2618	0.14	[0.13; 0.16]	2.1%	3.7%
**Fixed effect model**		**185258**	**0.10**	**[0.10; 0.10]**	**100%**	--
**Random effects model**			**0.11**	**[0.10; 0.13]**	--	**100%**

Heterogeneity: I-squared=98.7%, tau-squared=0.1691, p<0.0001

Meta-analysis of syphilis prevalence in among MSM in Northeast China

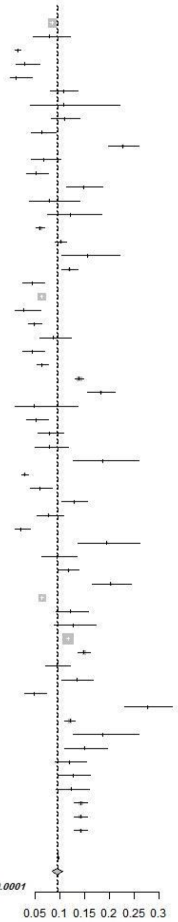

Study	Events	Total	Proportion	95%-CI	W(fixed)	W(random)
C1	2924	34292	0.09	[0.08; 0.09]	16.1%	2.0%
C108	18	228	0.08	[0.05; 0.12]	0.1%	1.5%
C112	24	1542	0.02	[0.01; 0.02]	0.1%	1.6%
C113	8	260	0.03	[0.01; 0.06]	0.0%	1.1%
C12	2	158	0.01	[0.00; 0.04]	0.0%	0.5%
C122	56	520	0.11	[0.08; 0.14]	0.3%	1.8%
C123	6	55	0.11	[0.04; 0.22]	0.0%	0.9%
C133	54	490	0.11	[0.08; 0.14]	0.3%	1.8%
C136	26	401	0.06	[0.04; 0.09]	0.1%	1.6%
C137	171	750	0.23	[0.20; 0.26]	0.8%	1.9%
C14	21	306	0.07	[0.04; 0.10]	0.1%	1.5%
C142	24	452	0.05	[0.03; 0.08]	0.1%	1.6%
C145	60	404	0.15	[0.12; 0.19]	0.3%	1.8%
C146	10	126	0.08	[0.04; 0.14]	0.1%	1.2%
C148	19	155	0.12	[0.08; 0.18]	0.1%	1.5%
C149	194	3164	0.06	[0.05; 0.07]	1.1%	1.9%
C150	297	2892	0.10	[0.09; 0.11]	1.6%	2.0%
C153	26	165	0.16	[0.11; 0.22]	0.1%	1.6%
C156	193	1600	0.12	[0.11; 0.14]	1.0%	1.9%
C157	18	403	0.04	[0.03; 0.07]	0.1%	1.5%
C164	2189	34100	0.06	[0.06; 0.07]	12.3%	2.0%
C166	6	208	0.03	[0.01; 0.06]	0.0%	1.0%
C174	54	1093	0.05	[0.04; 0.06]	0.3%	1.8%
C176	29	329	0.09	[0.06; 0.12]	0.2%	1.6%
C182	18	403	0.04	[0.03; 0.07]	0.1%	1.5%
C183	114	1752	0.07	[0.05; 0.08]	0.6%	1.9%
C2	854	6101	0.14	[0.13; 0.15]	4.4%	2.0%
C22	145	790	0.18	[0.16; 0.21]	0.7%	1.9%
C33	3	61	0.05	[0.01; 0.14]	0.0%	0.6%
C38	23	436	0.05	[0.03; 0.08]	0.1%	1.6%
C47	38	474	0.08	[0.06; 0.11]	0.2%	1.7%
C48	22	274	0.08	[0.05; 0.12]	0.1%	1.5%
C6	27	144	0.19	[0.13; 0.26]	0.1%	1.6%
C71	79	2680	0.03	[0.02; 0.04]	0.5%	1.9%
C74	31	506	0.06	[0.04; 0.09]	0.2%	1.7%
C76	89	687	0.13	[0.11; 0.16]	0.5%	1.9%
C8	33	421	0.08	[0.05; 0.11]	0.2%	1.7%
C83	9	403	0.02	[0.01; 0.04]	0.1%	1.2%
C90	33	169	0.20	[0.14; 0.26]	0.2%	1.6%
C91	28	294	0.10	[0.06; 0.13]	0.2%	1.6%
E100	121	1019	0.12	[0.10; 0.14]	0.6%	1.9%
E102	84	413	0.20	[0.17; 0.25]	0.4%	1.8%
E103	2189	33684	0.06	[0.06; 0.07]	12.3%	2.0%
E104	52	424	0.12	[0.09; 0.16]	0.3%	1.8%
E106	33	259	0.13	[0.09; 0.17]	0.2%	1.7%
E11	5552	47231	0.12	[0.11; 0.12]	29.4%	2.0%
E112	442	2958	0.15	[0.14; 0.16]	2.3%	2.0%
E115	56	587	0.10	[0.07; 0.12]	0.3%	1.8%
E17	64	475	0.13	[0.11; 0.17]	0.3%	1.8%
E20	21	428	0.05	[0.03; 0.07]	0.1%	1.5%
E25	98	352	0.28	[0.23; 0.33]	0.4%	1.8%
E27	466	3834	0.12	[0.11; 0.13]	2.5%	2.0%
E35	27	144	0.19	[0.13; 0.26]	0.1%	1.6%
E36	42	280	0.15	[0.11; 0.20]	0.2%	1.7%
E58	50	416	0.12	[0.09; 0.16]	0.3%	1.8%
E79	53	417	0.13	[0.10; 0.16]	0.3%	1.8%
E82	49	397	0.12	[0.09; 0.16]	0.3%	1.8%
E86	420	2936	0.14	[0.13; 0.16]	2.2%	2.0%
E95	422	2958	0.14	[0.13; 0.16]	2.2%	2.0%
E99	375	2618	0.14	[0.13; 0.16]	1.9%	2.0%
**Fixed effect model**		**201518**	**0.10**	**[0.10; 0.10]**	**100%**	--
**Random effects model**			**0.10**	**[0.09; 0.11]**	--	**100%**

Heterogeneity: I-squared=97.6%, tau-squared=0.1678, p<0.0001

Meta-analysis of syphilis prevalence in among MSM in East China

Study	Events	Total	Proportion	95%-CI	W(fixed)	W(random)
C1	2924	34292	0.09	[0.08; 0.09]	17.0%	3.3%
C11	36	251	0.14	[0.10; 0.19]	0.2%	2.8%
C130	8	1038	0.01	[0.00; 0.02]	0.1%	1.9%
C164	2189	34100	0.06	[0.06; 0.07]	13.0%	3.3%
C17	213	2889	0.07	[0.06; 0.08]	1.3%	3.2%
C175	44	922	0.05	[0.03; 0.06]	0.3%	2.9%
C177	288	2450	0.12	[0.11; 0.13]	1.6%	3.3%
C2	854	6101	0.14	[0.13; 0.15]	4.7%	3.3%
C24	17	366	0.05	[0.03; 0.07]	0.1%	2.4%
C40	33	341	0.10	[0.07; 0.13]	0.2%	2.8%
C43	28	458	0.06	[0.04; 0.09]	0.2%	2.7%
C63	34	405	0.08	[0.06; 0.12]	0.2%	2.8%
C65	44	820	0.05	[0.04; 0.07]	0.3%	2.9%
C7	13	911	0.01	[0.01; 0.02]	0.1%	2.2%
C81	83	2527	0.03	[0.03; 0.04]	0.5%	3.1%
C89	63	743	0.08	[0.07; 0.11]	0.4%	3.0%
C93	40	469	0.09	[0.06; 0.11]	0.2%	2.8%
C98	7	109	0.06	[0.03; 0.13]	0.0%	1.7%
E103	2189	33684	0.06	[0.06; 0.07]	13.0%	3.3%
E107	16	391	0.04	[0.02; 0.07]	0.1%	2.4%
E11	5552	47231	0.12	[0.11; 0.12]	31.1%	3.3%
E112	442	2958	0.15	[0.14; 0.16]	2.4%	3.3%
E27	466	3834	0.12	[0.11; 0.13]	2.6%	3.3%
E28	26	510	0.05	[0.03; 0.07]	0.2%	2.7%
E3	93	1000	0.09	[0.08; 0.11]	0.5%	3.1%
E33	90	568	0.16	[0.13; 0.19]	0.5%	3.1%
E53	144	513	0.28	[0.24; 0.32]	0.7%	3.2%
E71	166	1390	0.12	[0.10; 0.14]	0.9%	3.2%
E78	26	503	0.05	[0.03; 0.07]	0.2%	2.7%
E8	54	561	0.10	[0.07; 0.12]	0.3%	3.0%
E80	146	1692	0.09	[0.07; 0.10]	0.8%	3.2%
E86	420	2936	0.14	[0.13; 0.16]	2.3%	3.3%
E95	422	2958	0.14	[0.13; 0.16]	2.3%	3.3%
E99	375	2618	0.14	[0.13; 0.16]	2.0%	3.3%
**Fixed effect model**		**192539**	**0.10**	**[0.09; 0.10]**	**100%**	--
**Random effects model**			**0.08**	**[0.07; 0.10]**	--	**100%**

Heterogeneity: I-squared=98.4%, tau-squared=0.1554, p<0.0001

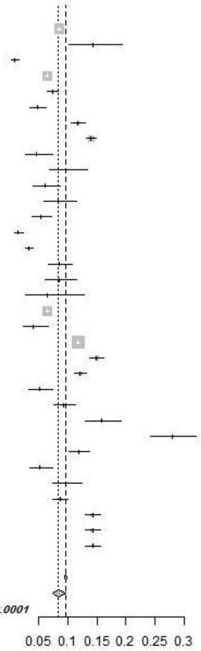

Meta-analysis of syphilis prevalence in among MSM in Southwest China

Study	Events	Total	Proportion	95%-CI	W(fixed)	W(random)
C1	2924	34292	0.09	[0.08; 0.09]	14.6%	2.3%
C100	26	248	0.10	[0.07; 0.15]	0.1%	2.0%
C101	30	393	0.08	[0.05; 0.11]	0.2%	2.0%
C107	127	507	0.25	[0.21; 0.29]	0.5%	2.2%
C132	40	452	0.09	[0.06; 0.12]	0.2%	2.1%
C138	86	800	0.11	[0.09; 0.13]	0.4%	2.2%
C144	8	404	0.02	[0.01; 0.04]	0.0%	1.5%
C159	30	392	0.08	[0.05; 0.11]	0.2%	2.0%
C164	2189	34100	0.06	[0.06; 0.07]	11.2%	2.3%
C168	799	14268	0.06	[0.05; 0.06]	4.1%	2.3%
C171	214	4904	0.04	[0.04; 0.05]	1.1%	2.3%
C173	90	603	0.15	[0.12; 0.18]	0.4%	2.2%
C2	854	6101	0.14	[0.13; 0.15]	4.0%	2.3%
C3	36	790	0.05	[0.03; 0.06]	0.2%	2.1%
C30	62	1432	0.04	[0.03; 0.06]	0.3%	2.2%
C31	53	462	0.11	[0.09; 0.15]	0.3%	2.1%
C39	11	89	0.12	[0.06; 0.21]	0.1%	1.6%
C4	6	155	0.04	[0.01; 0.08]	0.0%	1.3%
C42	37	287	0.13	[0.09; 0.17]	0.2%	2.1%
C45	232	2296	0.10	[0.09; 0.11]	1.1%	2.3%
C46	197	675	0.29	[0.26; 0.33]	0.8%	2.3%
C5	1097	5232	0.21	[0.20; 0.22]	4.7%	2.3%
C51	6	92	0.07	[0.02; 0.14]	0.0%	1.3%
C56	98	456	0.21	[0.18; 0.26]	0.4%	2.2%
C58	90	492	0.18	[0.15; 0.22]	0.4%	2.2%
C60	49	436	0.11	[0.08; 0.15]	0.2%	2.1%
C75	43	273	0.16	[0.12; 0.21]	0.2%	2.1%
C77	36	300	0.12	[0.09; 0.16]	0.2%	2.0%
C79	170	3445	0.05	[0.04; 0.06]	0.9%	2.3%
C84	47	476	0.10	[0.07; 0.13]	0.2%	2.1%
C9	99	400	0.25	[0.21; 0.29]	0.4%	2.2%
E103	2189	33684	0.06	[0.06; 0.07]	11.2%	2.3%
E11	5552	47231	0.12	[0.11; 0.12]	26.8%	2.3%
E112	442	2958	0.15	[0.14; 0.16]	2.1%	2.3%
E14	21	201	0.10	[0.07; 0.16]	0.1%	1.9%
E27	466	3834	0.12	[0.11; 0.13]	2.2%	2.3%
E31	59	1146	0.05	[0.04; 0.07]	0.3%	2.2%
E38	122	621	0.20	[0.17; 0.23]	0.5%	2.2%
E41	100	408	0.25	[0.20; 0.29]	0.4%	2.2%
E42	244	1376	0.18	[0.16; 0.20]	1.1%	2.3%
E65	40	388	0.10	[0.07; 0.14]	0.2%	2.1%
E71	166	1390	0.12	[0.10; 0.14]	0.8%	2.3%
E73	162	801	0.20	[0.17; 0.23]	0.7%	2.2%
E77	44	850	0.05	[0.04; 0.07]	0.2%	2.1%
E86	420	2936	0.14	[0.13; 0.16]	2.0%	2.3%
E95	422	2958	0.14	[0.13; 0.16]	2.0%	2.3%
E99	375	2618	0.14	[0.13; 0.16]	1.8%	2.3%
**Fixed effect model**		**218652**	**0.10**	**[0.10; 0.10]**	**100%**	--
**Random effects model**			**0.11**	**[0.10; 0.13]**	--	**100%**

Heterogeneity: I-squared=98.8%, tau-squared=0.2357, p<0.0001

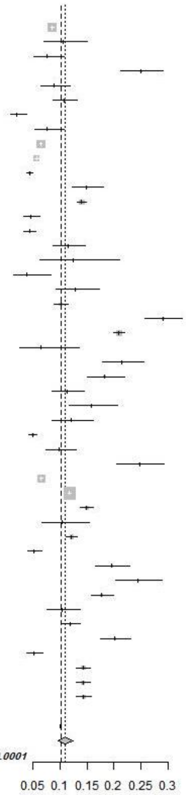

Meta-analysis of syphilis prevalence in among MSM in Southcentral China

Study	Events	Total	Proportion	95%-CI	W(fixed)	W(random)
C1	2924	34292	0.09	[0.08; 0.09]	17.3%	3.8%
C10	184	2074	0.09	[0.08; 0.10]	1.1%	3.7%
C109	74	492	0.15	[0.12; 0.19]	0.4%	3.5%
C110	47	400	0.12	[0.09; 0.15]	0.3%	3.3%
C115	32	407	0.08	[0.05; 0.11]	0.2%	3.2%
C116	138	806	0.17	[0.15; 0.20]	0.7%	3.6%
C126	41	400	0.10	[0.07; 0.14]	0.2%	3.3%
C134	71	324	0.22	[0.18; 0.27]	0.4%	3.4%
C139	92	401	0.23	[0.19; 0.27]	0.5%	3.5%
C140	171	802	0.21	[0.19; 0.24]	0.9%	3.6%
C154	29	475	0.06	[0.04; 0.09]	0.2%	3.1%
C164	2189	34100	0.06	[0.06; 0.07]	13.2%	3.8%
C173	90	603	0.15	[0.12; 0.18]	0.5%	3.5%
C180	29	3200	0.01	[0.01; 0.01]	0.2%	3.2%
C2	854	6101	0.14	[0.13; 0.15]	4.7%	3.8%
C49	105	1172	0.09	[0.07; 0.11]	0.6%	3.6%
C70	29	475	0.06	[0.04; 0.09]	0.2%	3.1%
E103	2189	33684	0.06	[0.06; 0.07]	13.2%	3.8%
E11	5552	47231	0.12	[0.11; 0.12]	31.6%	3.8%
E112	442	2958	0.15	[0.14; 0.16]	2.4%	3.7%
E16	42	229	0.18	[0.14; 0.24]	0.2%	3.2%
E27	466	3834	0.12	[0.11; 0.13]	2.6%	3.7%
E71	166	1390	0.12	[0.10; 0.14]	0.9%	3.7%
E74	66	463	0.14	[0.11; 0.18]	0.4%	3.4%
E83	2	215	0.01	[0.00; 0.03]	0.0%	1.0%
E84	73	218	0.33	[0.27; 0.40]	0.3%	3.4%
E86	420	2936	0.14	[0.13; 0.16]	2.3%	3.7%
E95	422	2958	0.14	[0.13; 0.16]	2.3%	3.7%
E99	375	2618	0.14	[0.13; 0.16]	2.1%	3.7%
**Fixed effect model**		**185258**	**0.10**	**[0.10; 0.10]**	**100%**	--
**Random effects model**			**0.11**	**[0.10; 0.13]**	--	**100%**

Heterogeneity: I-squared=98.7%, tau-squared=0.1691, p<0.0001

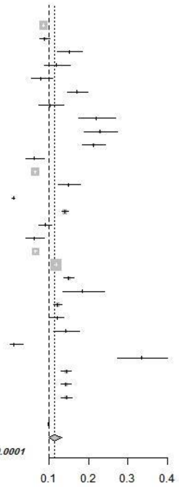

Meta-analysis of syphilis prevalence in among MSM in Northwest China
